# Political ideology and pandemic lifestyles: the indirect effects of empathy, authoritarianism, and threat

**DOI:** 10.1007/s44155-022-00014-0

**Published:** 2022-08-24

**Authors:** Terrence D. Hill, Ginny Garcia-Alexander, Andrew P. Davis, Eric T. Bjorklund, Luis A. Vila-Henninger, William C. Cockerham

**Affiliations:** 1 Department of Sociology, University of Texas at San Antonio, One UTSA Circle, San Antonio, TX 78249 USA; 2 Department of Sociology & Anthropology, North Carolina State University, Raleigh, NC USA; 3 School of Social and Family Dynamics, Arizona State University, Tempe, AZ USA; 4 Department of Political Science, Aarhus University, Aarhus, Denmark; 5 Departments of Sociology, University of Alabama at Birmingham and College of William & Mary, Birmingham, AL USA

**Keywords:** Political ideology, Political conservatism, Pandemic lifestyles, Empathy, Authoritarianism, Pandemic threat

## Abstract

**Background:**

In this paper, we integrate theory and research from sociology, psychology, and political science to develop and test a mediation model that helps to explain *why* political conservatism is often associated with pandemic behaviors and lifestyles that are inconsistent with public health recommendations for COVID-19.

**Methods:**

Using national data from the 2021 *Crime, Health, and Politics Survey* (n = 1743), we formally test the indirect effects of political conservatism (an index of Republican party identification, conservative political orientation, right-wing news media consumption, and 2020 Trump vote) on pandemic lifestyles (an index of social distancing, hand sanitizing, mask usage, and vaccination) through the mechanisms of empathy (concern about the welfare of others), authoritarian beliefs (authoritarian aggressiveness and acquiescence to authority), and pandemic threat perceptions (threats to self and to the broader society).

**Result:**

Our results confirm that political conservatism is associated with riskier pandemic lifestyles. We also find that this association is partially mediated by lower levels of empathy, higher levels of authoritarian beliefs, and lower levels of perceived pandemic threat.

**Conclusions:**

Understanding *why* political conservatism is associated with riskier pandemic lifestyles may eventually lead us to ways of identifying and overcoming widespread cultural barriers to critical pandemic responses.

## Introduction

Over the course of the novel coronavirus (COVID-19) pandemic, several studies have linked various indicators of political conservatism (conservative political identity and ideology) with riskier pandemic health behaviors (specific health behaviors that are relevant to the transmission of infectious pathogens) and more precarious pandemic health lifestyles (the clustering of pandemic health behaviors within individuals and populations). At the ecological-level, research shows that Republican-leaning states and counties that favored Trump in the 2016 and 2020 Presidential elections tend to exhibit lower rates of sheltering-in-place, social distancing, mask usage, and COVID-19 vaccination [1–8]. At the individual-level, studies confirm that people who identify as Trump voters, Republicans, or political conservatives tend to report generally insalubrious pandemic lifestyles, including lower rates of social distancing, hand sanitizing, mask usage, and vaccination [1, 9–18].

Although these studies are seminal in the sense that they extend established chronic disease models (e.g., of smoking and drinking) that traditionally exclude political variables [18], the theoretical and empirical mechanisms underlying the effects of political conservatism remain understudied [19]. In a recent PBS interview, Dr. Francis Collins, the director of the National Institutes of Health (NIH), lamented that the NIH has “underinvested in research on human behavior” and that he “never imagined” that “we would still have 60 million people who had not taken advantage of [safe and effective vaccines] because of misinformation and disinformation…” [20]. In this paper, we contribute to the social epidemiology of infectious disease behavior by developing and testing a mediation model to help explain *why* political conservatism is often associated with riskier pandemic lifestyles.

## Theoretical model

Our theoretical model proposes that political conservatism contributes to risky pandemic lifestyles through the mechanisms of empathy, authoritarianism, and pandemic threat perceptions. Our model is informed by the integration of theory and research from sociology, psychology, and political science. In this section, we explore the theoretical and empirical basis for each link in our proposed model. While pandemic threat perceptions have received a great deal of attention [1, 11, 13–15, 17], concepts related to empathy [9, 12] and authoritarian beliefs [21, 22] have not. Moreover, to our knowledge, researchers have only begun to formally test any mechanisms of political variations in pandemic lifestyles [17]. We note at the outset that our proposed model should be viewed as one viable model with compelling theoretical alternatives. While we focus on our model in this section, we consider some theoretical alternatives later in the discussion section.

### Health lifestyle theory

Cockerham et al. [23, 24] developed health lifestyle theory to challenge the dominant notion in public health of health lifestyles as “uncoordinated practices of disconnected individuals.” Cockerham et al. [23, p. 321] defined health lifestyles as “collective patterns of health-related behavior based on choices from options available to people according to their life chances” to emphasize the dual forces of agency and structure. Although health lifestyle theory has many facets that are beyond the scope of our investigation, we want to highlight the primary role of “collectivities” in Cockerham’s paradigm. Cockerham [24, p. 59] explains that “collectivities are collections of actors linked together through particular social relationships, such as kinship, work, religion, and politics” whose “shared norms, values, ideals, and social perspectives constitute intersubjective ‘thought communities’ beyond individual subjectivity that reflect a particular collective world view.” The recurring interplay of choices (agency) and chances (structure) through socialization and experience leads to the development of habitus or internalized dispositions toward behavior or action. According to Cockerham [24, p. 61], “the habitus serves as a cognitive map or set of perceptions that routinely guides and evaluates a person’s choices and options. It provides enduring dispositions toward acting deemed appropriate by a person in particular social situations and settings.” Once the habitus is formed, health-related practices like smoking, exercise, and dietary behavior become “integrated into routine behavioral repertories that can be acted out more or less unthinkingly” [24, p. 62].

Our proposed model flows from health lifestyle theory in the sense that it frames pandemic lifestyles as outcomes of political socialization and experience. Our argument is that political collectivities drive the empathy, authoritarianism, and threat perceptions that proximally underlie the clustering of seemingly discrete pandemic health behaviors among individuals and populations. Our proposed model extends health lifestyle theory in three key respects. First, scholars have only recently applied health lifestyle theory to pandemic health behavior. Research along these lines is necessary because “*new health behaviors*—at least in the United States—have emerged…to slow the spread of the virus” [25, p. 923]. Second, we center political and ideological forces that have been conventionally overlooked in the health lifestyle literature [24, 26]. While there is at least some evidence linking pro-socialist ideology with passive health lifestyles in Russia [27], more recent work suggests that political distinctions can be causes *and* consequences of pandemic lifestyles in the United States [25]. Finally, we formally test several mechanisms linking political conservatism and health lifestyles. In a recent review of health lifestyles research, Mollborn et al. [26, p. 387] note that “the social psychological underpinnings of health lifestyles—including group-based norms, identities, social control, and understandings of health—must be more clearly articulated.”

### Empathy

The first link in our theoretical model suggests that political conservatism contributes to riskier pandemic lifestyles by reducing empathetic concern. While we are regularly inundated with popular depictions of “heartless conservatives” and “bleeding heart liberals,” research consistently shows that political conservatives tend to exhibit lower levels of compassion and empathy (i.e., less concern for the feelings and experiences of others) relative to their more liberal counterparts [28–35]. Cameron and Rapier [36, p. 391] explain that whereas “liberals tend to focus on the moral principle of care/harm [the ability to feel and to be disturbed by the pain of others], conservatives tend to emphasize individual responsibility, and these may constrain how compassion is expressed.” These tendencies are at least partially supported by differences in attribution styles. According to Hasson et al. [28, p. 1450], “liberals often attribute external causes to people’s plight (e.g., perceive unjust social practices and structures as causes of poverty) and feel more sympathy toward them, while conservatives attribute internal causes (e.g., perceive laziness and drug use as causes of poverty) and feel less sympathy.” There are also important ideological differences. On the one hand, liberals may be more empathetic and compassionate toward the suffering of others because they tend to emphasize “fairness and equality” and “justice” [29, p. 656]. On the other hand, conservatives may be less empathetic and compassionate because they are more concerned with “order and traditionalism” and “ingroup loyalty, respect for authority, and purity” [29, p. 656].

Political differences in empathy are not intrinsically important. Arguably, empathy is important insofar as it promotes prosocial behavior that is relevant to the health and well-being of others, including behavioral precautions during epidemics and pandemics [37–44]. The idea is that people who care about the feelings and experiences of others may be more likely to engage in lifestyles that favor public health during a pandemic by limiting viral transmission and protecting the health of vulnerable populations. There is some preliminary evidence to support this proposition. In their analysis of national survey data collected from 1015 adults, Motta and Goren [19] report that the basic human value of self-transcendence (concern for the welfare of others) is strongly associated with greater “pro-social health behavior” (higher scores on a comprehensive index of pandemic health behaviors). Accounting for the possibility of social desirability bias (from self-reports of health behavior), Motta and Goren [19] also demonstrate that states scoring higher on self-transcendence tend to engage in greater social distancing behavior (as indicated by objective cell phone-tracking). Using data collected from a Mechanical Turk (MTurk) sample of 2001 adults, Fazio et al. [12] show that respondents who report higher levels of (a) general empathy and (b) specific concern for the vulnerability of others in the context of COVID-19 tend to exhibit greater self-reported social distancing (past behavior) and virtual social distancing (real-time graphical scenarios). In another MTurk study of 1033 adults, Chan [9] reveals that respondents who score higher on the moral principle of care/harm (the ability to feel and to be disturbed by the pain of others) tend to report greater “compliance intentions” with respect to wearing face masks, staying at home, and social distancing. Based on these results, Chan [9, p. 7] suggests that public health campaigns should emphasize how engaging in healthy pandemic lifestyles can demonstrate that “one is caring and is fair for all members of society.”

### Authoritarian beliefs

The second link in our theoretical model suggests that political conservatism contributes to riskier pandemic lifestyles by encouraging authoritarian belief systems. In the macro sense of the term, “authoritarian regimes are characterized by, among other things, weakened institutions, the unregulated use of executive power, repression, and patronage with its concomitant loyalty to the ruler or ruling party” [44, p. 504]. In the micro sense, authoritarianism is a personality type that centers on the belief “that submission to authority is essential and that those who fail to submit are to be punished” [44, p. 504]. Although authoritarianism can be conceptualized in various ways [45–47], we are primarily interested in beliefs concerning authoritarian aggressiveness and acquiescence to authority. For authoritarians, punishment and submission are expressions of “a fear of uncertainty and the desire for social order” [48, p. 730]. Studies consistently show that political conservatives, Republican partisans, and Trump supporters tend to report higher levels of this micro-level form of authoritarianism [48–54]. Luttig [50, p. 786] explains that “as the GOP became more conservative on social issues, embraced the Religious Right, advocated being tough on crime, advanced a more Hawkish foreign policy, and signaled their opposition to illegal immigration, they communicated that their party sees the world as a dangerous place and that they value obedience, respect, good manners, and good behavior.”

Although authoritarianism has been linked with support for rigid pandemic-mitigation policies (e.g., “heads of national, state, and local governments should be able to order new restrictions on activities that could spread the virus, without needing to consult legislative bodies such as Congress or state legislatures”) [21, p. 3], there are compelling reasons why authoritarian beliefs might also be associated with the rejection of public health recommendations and riskier pandemic lifestyles [22]. Because people who hold authoritarian beliefs are especially receptive to the rhetoric of charismatic leaders, they are often motivated to rally against competing authorities, especially rational-bureaucratic “liberal” authorities [21, 44, 55]. Authoritarian beliefs are consistently associated with the rejection of science (a competing authority), including disagreement with scientific consensus (e.g., related to climate change, vaccination, and evolution) and general distrust of science and scientists [22, 56, 57]. This orientation enabled Trump and other prominent Republicans to downplay the threat of the pandemic, contradict the recommendations of public health organizations, and promote conspiracy theories and dubious pharmaceutical interventions [4, 6, 22]. Trump essentially forced public health and front-line medical professionals to openly challenge his commentary and authority. While lamenting the “politicization of public health,” Robert Faris, research director at Harvard’s Berkman Klein Center for Internet & Society, noted that “having Trump and Fauci on the same public stage at the same time [became] an untenable position…” [58]. The adversarial relationship between Trump (the charismatic leader) and institutions of public health and medicine (competing authorities) may help to explain the empirical link between authoritarian beliefs and risky pandemic health behaviors and attitudes. In their MTurk study of 200 adults, Prichard and Christman [22] show that respondents who score higher on authoritarianism are less likely to wear masks in public and less concerned about the impact of COVID-19 on their health and the health of others. In this context, Prichard and Christman [22, p. 6] question “whether politics have led people who endorse authoritarian views, in particular Right-wing Authoritarians, to shift away from being concerned about the virus.”

### Pandemic threat

The final link in our theoretical model suggests that political conservatism contributes to riskier pandemic lifestyles by reducing pandemic threat perceptions, including threats defined as personal/egocentric (the pandemic threatens my personal health and finances) and societal/sociotropic (the pandemic threatens public health and the economy). Studies and polls consistently show that political conservatives systematically minimize the severity of the pandemic [1, 13–15, 17, 59–65]. There are three primary explanations for these patterns. First, political conservatives tend to distrust “big government” because conservative rhetoric often frames the federal government as an ineffective and malignant “deep state” bureaucracy that exists to serve its own interests by extracting ever-increasing taxes and diminishing personal freedom and liberty [66–68]. Second, anti-intellectualism runs deep in American conservative politics and is commonly expressed through a suspicious and adversarial relationship vis-à-vis science in general and COVID science in particular [13, 15, 69–74]. Third, distrust of the Centers for Disease Control and Prevention and the World Health Organization has been fostered from the early stages of the pandemic when Trump, other political elites, and right-wing media openly challenged public health officials (e.g., Anthony Fauci and Robert Redfield) and repeatedly described the pandemic as a “fraud” perpetrated by the “deep state,” as “fake news,” as a “liberal hoax,” and as an “impeachment scam” [4, 14, 17, 75–77]. Taken together, these explanations suggest that political conservatives may be especially likely to question or oppose (a) pandemic policies and mandates set by the federal government, (b) the legitimacy of COVID science, and (c) the efficacy of public health recommendations (e.g., mask use and vaccinations).

Pandemic threat perceptions are potentially important as a source of motivation for healthier pandemic lifestyles. Allcott et al. [1, p. 9] speculate that “if Republicans and Democrats disagree about the potential risks [of the pandemic], they may also differ in how much they reduce the risk of disease transmission through social distancing and other actions.”

Invoking the theory of danger control, Roberto et al. [78, p. 265] argue that when pandemic threat perceptions and the perceived efficacy of public health mitigation strategies are high, “a person should experience greater protection motivation (i.e., intention) and ultimately adaptive changes (i.e., behavior).” Indeed, several studies show that people who report more concerns about the pandemic and greater personal vulnerability to COVID-19 are more likely to comply with public health recommendations for movement restrictions, social distancing, wearing masks, hand washing, and vaccination [9, 12, 60, 78–81]. There is also suggestive qualitative evidence. In a recent in-depth interview study of how families manage social distancing behaviors, a parent offered the following observation: “A lot of people not wearing masks. I don’t see them keeping their social distance… My perception is a lot of people don’t think it’s very serious…” [25, p. 930]. To this point, however, no studies have formally tested whether pandemic threat perceptions mediate the association between political conservatism and pandemic lifestyles.

### Hypotheses

In accordance with these arguments, we developed three mediation hypotheses to guide subsequent analyses. Hypothesis 1: The association between political conservatism and risky pandemic lifestyles will be mediated by lower levels of empathetic concern. Hypothesis 2: The association between political conservatism and risky pandemic lifestyles will be mediated by higher levels of authoritarian beliefs. Hypothesis 3: The association between political conservatism and risky pandemic lifestyles will be mediated by lower levels of perceived pandemic threat.

## Material and methods

### Data

We use data from the 2021 *Crime, Health, and Politics Survey* (CHAPS). The primary purpose of CHAPS is to document the social causes and social consequences of various indicators of health and well-being in the United States during the coronavirus (COVID-19) pandemic. CHAPS is based on a national probability sample of 1771 non-institutionalized adults aged 18 and over living in the United States. Respondents were sampled from the National Opinion Research Center’s (NORC) *AmeriSpeak*© panel, which is representative of households from all 50 states and the District of Columbia (https://amerispeak.norc.org/Documents/Research/AmeriSpeak%20Technical%20Overview%202019%2002%2018.pdf). Sampled respondents were invited to complete the online survey in English between May 10, 2021 and June 1, 2021. The data collection process yielded a survey completion rate of 30.7% and a weighted cumulative response rate of 4.4%. The weighted cumulative response rate, which considers all panel recruitment and retention rates, is the overall survey response rate that accounts for survey outcomes in all response stages, including the panel recruitment rate, panel retention rate, and survey completion rate. It is weighted to account for the sample design and differential inclusion probabilities of sample members. Our cumulative response rate is within the typical range (4–5%) of high-quality general population surveys (see https://www.pewresearch.org/politics/2021/05/17/scope-of-government-methodology/). The multistage probability sample resulted in a margin of error of ± 3.23% and an average design effect of 1.92. Margin of error is defined as half the width of the 95% confidence interval for a proportion estimate of 50% adjusted for design effect. A figure of ± 3.23% is therefore the largest margin of error possible for all estimated percentages based on the study sample. A margin of error of ± 3.23% at the 95% confidence level means that if we fielded the same survey 100 times, we would expect the result to be within 3.23% of the true population value 95 times. Although a margin of error of 3.00 is considered to be very good and in line with conventional practices, a smaller margin of error would be indicative of more precise estimates [82]. The average design effect is the variance under the complex design divided by the variance under a simple random sampling design of the same sample size. The design effect is variable-specific, and the reported value is the average design effect calculated for a set of key survey variables. Design effects account for deviations from simple random sampling with a 100% response rate. A design effect of 1.92 is very good because it means that the variance is only about twice as large as would be expected with simple random sampling [83]. The median self-administered web-based survey lasted approximately 25 min. All respondents were offered the cash equivalent of $8.00 for completing the survey, which is on the more lucrative end of the incentive spectrum for a survey of this duration. The survey was reviewed and approved by the institutional review board at NORC and one other university review board. Informed consent was obtained from all participants.

### Measures

*Political conservatism* We infer political conservatism with a four-part composite measure of partisan identification, ideological orientation, political consumption patterns, and political behavior to better capture the multi-faceted and dynamic nature of contemporary politically engagement in America. More specifically, we measure political conservatism as the mean response to four items: (a) Republican party identification (1 = Republican; 0 = otherwise); (b) conservative political orientation (1 = very liberal to 5 = very conservative); (c) right-wing news media consumption for current events (1 = Fox News, Newsmax, One America New Network, Alex Jones, Drudge Report, Breitbart, Infowars, and the like; 0 = ABC, CBS, CNN, MSNBC, NBC, and the like); and (d) Trump vote in the 2020 Presidential election (1 = yes; 0 = no). To account for metric differences, each of these items was standardized before indexing. All items were coded so that higher values would indicate greater political conservatism. An exploratory principal components analysis with varimax rotation produced a single component (eigenvalue = 2.67), with loadings ranging from 0.72 to 0.88. A reliability analysis suggested adequate internal consistency for four items (α = 0.83).

*Pandemic lifestyles* We measure pandemic lifestyles as the mean response to four items. Respondents were asked to indicate how often during the coronavirus (COVID-19) pandemic they (a) “attended indoor gatherings with more than 10 people,” (b) “used hand sanitizer to kill germs after being in public places, and (c) wore “a mask or other face covering in public places.” Responses to these items ranged from (1) never to (5) always, with reverse-coding for indoor gatherings. Respondents were also asked whether they (d) had been “vaccinated for the coronavirus (COVID-19).” Responses to this item were coded (1) yes and (0) no. To account for metric differences, each of these items was standardized before indexing. All items were coded so that higher values would indicate healthier pandemic lifestyles. An exploratory principal components analysis with varimax rotation produced a single component (eigenvalue = 1.97), with loadings ranging from 0.65 to 0.83. A reliability analysis suggested adequate internal consistency for four items (α = 0.65).

*Empathic concern* We measure empathic concern as the mean response to three items [84]. Respondents were asked to indicate the extent to which they agree or disagree with the following statements: (a) “I am often concerned about people less fortunate than me.” (b) “I often feel sorry for people when they are having problems in their lives.” (c) “I often feel protective towards people who are being taken advantage of.” Responses to these items ranged from (1) strongly disagree to (5) strongly agree. An exploratory principal components analysis with varimax rotation produced a single component (eigenvalue = 2.17), with loadings ranging from 0.84 to 0.86. A reliability analysis suggested adequate internal consistency for three items (α = 0.81).

*Authoritarian beliefs* We measure beliefs concerning authoritarian aggressiveness and acquiescence to authority as the mean response to three items. Respondents were asked to indicate the extent to which they agree or disagree with the following statements: (a) “What our country really needs is a tough, harsh dose of law and order.” (b) “The government would be justified in using violence to eliminate the troublemakers in this country to get us back on track.” (c) “Our country would be better off with a strong leader who did not have to bother with democracy and elections.” The first two items were drawn from previous research [47]. The last item was inspired by a similar item from the *World Values Survey* (“Having a strong leader who does not have to bother with parliament and elections”). Responses to these items ranged from (1) strongly disagree to (5) strongly agree. An exploratory principal components analysis with varimax rotation produced a single component (eigenvalue = 2.00), with loadings ranging from 0.75 to 0.87. A reliability analysis also suggested adequate internal consistency for three items (α = 0.75).

*Pandemic threat* We measure perceived pandemic threat as the mean response to four items and four two-item mean indices. Respondents were asked to indicate the extent to which they agree or disagree with the following statements about the coronavirus (COVID-19) pandemic: (a) “The coronavirus pandemic is a major threat to public health in the United States.” (b) “The coronavirus pandemic is a major threat to your personal health.” (c) “The coronavirus pandemic is a major threat to the economy in the United States.” (d) “The coronavirus pandemic is a major threat to your personal financial situation.” Responses to these items ranged from (1) strongly disagree to (5) strongly agree. All items were coded so that higher values would indicate greater pandemic threat. An exploratory principal components analysis with varimax rotation produced a single component (eigenvalue = 2.37), with loadings ranging from 0.67 to 0.86. A reliability analysis suggested adequate internal consistency for four items (α = 0.77). We also consider specific pandemic threats to health (items a and b, α = 0.85), economic status (items c and d, α = 0.56), self or personal health/economic status (items b and d, α = 0.62), and society or public health/economy (items a and c, α = 0.60).

*Background variables* Background variables include *age* (continuous years), *sex* (1 = female; 0 = male), *race/ethnicity* (dummy variables for non-Hispanic Black, Latino, and other race or ethnicity, with non-Hispanic White serving as the reference), *nativity status* (1 = US-born; 0 = otherwise), *southern residence* (1 = South; 0 = otherwise), *rural residence* (1 = rural; 0 = otherwise); *college degree* (1 = four-year college degree or higher; 0 = otherwise), *employment* (1 = employed; 0 = otherwise), *annual household income* (1 =  < $10,000 to 9 =  ≥ $150,000), *financial strain* (mean response to three items assessing the extent to which the respondent has trouble paying for health care, monthly bills, and food, α = 0.89), *marital status* (1 = married; 0 = otherwise), *children* (1 = presence of a child under age 18; 0 = otherwise), and *religiosity* (mean response to four items assessing in-person and virtual religious attendance, religious importance, and frequency of prayer, α = 0.85).

### Analysis

Due to listwise deletion of missing data, our analytic sample size was reduced from 1771 to 1743. Post-stratification weights were used in supplemental analyses to assess sampling error and non-response bias. NORC developed post-stratification weights for CHAPS via iterative proportional fitting or raking to general population parameters derived from the *Current Population Survey* (https://www.census.gov/programs-surveys/cps/data.html). These parameters included age, sex, race/ethnicity, education, and several interactions (age*sex, age*race, and sex*race).

Table 1 presents descriptive statistics for all study variables, including variable ranges, sample means, and standard deviations. In Table 2, we use ordinary least squares (OLS) regression to model empathic concern, authoritarian beliefs, and perceived pandemic threat as a function of political conservatism and background variables. In Table 3, we use OLS to model pandemic lifestyles as a function of political conservatism, mediators, and background variables. In Table 4, we model our mediators and pandemic lifestyles as a function of political conservatism items and background variables to assess the unique contribution of each item. All regression models in Tables 2, 3, 4 present standardized OLS coefficients and two-tailed statistical tests.

**Table 1:** Descriptive statistics (*CHAPS* 2021)

	Range	Mean	Standard deviation
Political Conservatism	− 1.13 to 1.57	− 0.003	0.81
Pandemic Lifestyles	− 2.67 to 0.84	− 0.002	0.70
Empathic Concern	1 to 5	3.99	0.66
Authoritarian Beliefs	1 to 5	2.40	0.97
Pandemic Threat	1 to 5	3.62	0.86
Pandemic Threat (Health)	1 to 5	3.58	1.11
Pandemic Threat (Economic)	1 to 5	3.66	0.87
Pandemic Threat (Self)	1 to 5	3.27	1.02
Pandemic Threat (Society)	1 to 5	3.98	0.88
Age	18 to 94	49.67	17.00
Female	0 to 1	0.52	
Non-Hispanic White	0 to 1	0.67	
Non-Hispanic Black	0 to 1	0.11	
Latino	0 to 1	0.16	
Other Race/Ethnicity	0 to 1	0.06	
US-Born	0 to 1	0.92	
Southern Residence	0 to 1	0.33	
Rural Residence	0 to 1	0.16	
College Degree	0 to 1	0.36	
Employed	0 to 1	0.62	
Household Income	1 to 9	5.56	2.23
Financial Strain	1 to 5	1.66	0.91
Married	0 to 1	0.54	
Presence of Children	0 to 1	0.17	
Religiosity	− 1.06 to 1.84	0.00	0.83

n = 1743

**Table 2:** Regression of empathic concern, authoritarianism, and pandemic threat (*CHAPS* 2021)

	Empathic concern	Authoritarian beliefs	Pandemic threat	Threat (Health)	Threat (Economic)	Threat (Self)	Threat (Society)
Political Conservatism	− 0.27***	0.45***	− 0.36***	− 0.46***	− 0.13***	− 0.29***	− 0.36***
Age	0.03	− 0.02	0.16***	0.19***	0.07**	0.15***	0.14***
Female	0.11***	0.04	0.004	0.01	− 0.01	0.01	0.00
Non-Hispanic Black	− 0.08**	0.12***	0.06**	0.06**	0.05	0.07**	0.04
Latino	− 0.07**	0.08**	0.04	0.04	0.02	0.06**	0.003
Other Race/Ethnicity	− 0.03	0.05*	0.03	0.02	0.04	0.06*	− 0.003
US-Born	0.02	− 0.05*	− 0.02	− 0.02	− 0.02	− 0.04	0.004
Southern Residence	0.03	− 0.01	0.02	0.03	0.01	0.04	− 0.01
Rural Residence	0.03	0.02	0.01	0.01	0.01	0.02	− 0.002
College Degree	0.02	− 0.17***	0.04	0.05*	0.01	− 0.01	0.09***
Employed	− 0.05*	− 0.01	− 0.02	− 0.03	− 0.001	− 0.02	− 0.02
Household Income	0.01	− 0.06*	− 0.02	− 0.02	− 0.02	− 0.04	0.001
Financial Strain	0.07**	0.04	0.15***	0.05*	0.22***	0.23***	0.02
Married	0.01	0.05*	− 0.002	− 0.01	0.02	0.01	− 0.01
Presence of Children	− 0.02	0.02	− 0.02	− 0.03	− 0.004	0.01	− 0.06*
Religiosity	0.22***	− 0.02	0.03	0.03	0.02	0.04	0.01
R-Squared	0.12	0.26	0.18	0.25	0.08	0.18	0.16

n = 1743. Shown are standardized OLS regression coefficients (*p < 0.05, **p < 0.01, ***p < 0.001)

**Table 3:** Regression of healthy pandemic lifestyles (*CHAPS* 2021)

	Model 1	Model 2	Model 3	Model 4	Model 5	Model 6	Model 7	Model 8	Model 9
Political Conservatism	− 0.41***	− 0.37***	− 0.37***	− 0.27***	− 0.20***	− 0.38***	− 0.32***	− 0.26***	− 0.21***
Empathetic Concern		0.13***							0.05*
Authoritarian Beliefs			− 0.10***						− 0.10***
Pandemic Threat				0.39***					0.39***
Threat (Health)					0.45***				
Threat (Economic)						0.21***			
Threat (Self)							0.31***		
Threat Society)								0.41***	
Age	0.27***	0.27***	0.27***	0.21***	0.18***	0.25***	0.22***	0.21***	0.21***
Female	0.08***	0.07**	0.09***	0.08***	0.08***	0.08***	0.08***	0.08***	0.08***
Non-Hispanic Black	0.01	0.02	0.03	− 0.01	− 0.01	0.03	− 0.01	− 0.01	0.01
Latino	0.02	0.03	0.03	0.01	0.02	0.02	0.02	0.02	0.02
Other Race/Ethnicity	0.01	0.01	0.01	− 0.04	− 0.01	0.01	− 0.01	0.01	0.02
US-Born	− 0.04*	− 0.05*	− 0.05*	− 0.04*	− 0.04	− 0.04	− 0.03	− 0.05*	− 0.04*
Southern Residence	− 0.02	− 0.02	− 0.02	− 0.02	− 0.03	− 0.02	− 0.03	− 0.01	− 0.03
Rural Residence	− 0.05*	− 0.05*	− 0.04*	− 0.05**	− 0.05**	− 0.05*	− 0.05**	− 0.04*	− 0.05**
College Degree	0.07**	0.07**	0.05**	0.05*	0.04*	0.07**	0.07***	0.03	0.04
Employed	− 0.04	− 0.04	− 0.04	− 0.03	− 0.03	− 0.04	− 0.04	− 0.03	− 0.03
Household Income	0.04	0.03	0.03	0.04*	0.04*	0.04	0.05*	0.04	0.04
Financial Strain	− 0.05*	− 0.05*	− 0.04*	− 0.10***	− 0.07***	− 0.09***	− 0.12***	− 0.05**	− 0.10***
Married	0.02	0.02	0.03	0.02	0.03	0.02	0.02	0.03	0.02
Presence of Children	− 0.07**	− 0.07**	− 0.06**	− 0.06**	− 0.05**	− 0.07**	− 0.07***	− 0.05*	− 0.06**
Religiosity	− 0.01	− 0.04	− 0.01	− 0.02	− 0.02	− 0.02	− 0.02	− 0.02	− 0.04
R-Squared	0.27	0.29	0.29	0.40	0.43	0.32	0.35	0.41	0.41
Nested F		34.08***	17.05***	363.34***	469.47***	106.99***	204.50***	404.82***	133.51***

n = 1743. Shown are standardized OLS regression coefficients (*p < 0.05, **p < 0.01, ***p < 0.001). Nested F statistics compare the fit of Model 1 with the fit of Models 2–9

**Table 4:** Regression of mediators and healthy pandemic lifestyles on political conservatism items (*CHAPS* 2021)

	Empathetic concern	Authoritarian beliefs	Pandemic threat	Threat (Health)	Threat (Economic)	Threat (Self)	Threat (Society)	Pandemic Lifestyles
Republican	− 0.16***	0.16***	− 0.15***	− 0.17***	− 0.08*	− 0.13***	− 0.15***	− 0.16***
Conservative Orientation	− 0.42***	0.19***	− 0.14***	− 0.15***	− 0.08**	− 0.11***	− 0.15***	− 0.13***
Right-Wing Media	− 0.03	0.07**	− 0.02	− 0.04	0.01	− 0.01	− 0.02	− 0.02
Trump Voter	− 0.02	0.13***	− 0.14***	− 0.19***	− 0.02	− 0.11**	− 0.14***	− 0.19***
R-Squared	0.13	0.28	0.19	0.27	0.08	0.19	0.18	0.30

We use conditional process analysis to formally test our mediation model [85]. In Table 5, we present the direct and simple indirect effects of political conservatism on pandemic lifestyles. Direct effects are shown as unstandardized OLS coefficients and two-tailed statistical tests. Simple indirect effects are tested with 95% bootstrap confidence intervals obtained from 10,000 bootstrap samples. Bootstrap confidence intervals are preferable to normal theory-based mediation tests because indirect effects (products of component paths) are not normally distributed [85]. Table 5 also provides pairwise contrasts of indirect effects to directly test whether one indirect effect is statistically different from another.

**Table 5:** Direct and indirect effects of political conservatism on healthy pandemic lifestyles

	Direct effects of political conservatism	Simple indirect effects	Pairwise contrasts of indirect effects
Conservatism → Empathy → Lifestyles	− 0.32***	− 0.03* (− 0.04, − 0.02)	
Conservatism → Authoritarian Beliefs → Lifestyles	− 0.32***	− 0.04* (− 0.06, − 0.02)	
Conservatism → Threat → Lifestyles	− 0.32***	− 0.12* (− 0.14, − 0.10)	
Conservatism → Threat (Health) → Lifestyles	− 0.18***	− 0.18* (− 0.21, − 0.15)	
Conservatism → Threat (Economic) → Lifestyles	− 0.33***	− 0.02* (− 0.03, − 0.01)	
Conservatism → Threat (Self) → Lifestyles	− 0.28***	− 0.08* (− 0.09, − 0.06)	
Conservatism → Threat (Society) → Lifestyles	− 0.23***	− 0.13* (− 0.15, − 0.11)	
Empathy vs. Authoritarian Beliefs			0.003 (− 0.02, 0.03)
Empathy vs. Pandemic Threat			− 0.10* (− 0.13, − 0.08)
Empathy vs. Threat (Health)			− 0.16* (− 0.19, − 0.13)
Empathy vs. Threat (Economic)			− 0.001 (− 0.02, 0.01)
Empathy vs. Threat (Self)			− 0.05* (− 0.07, − 0.03)
Empathy vs. Threat (Society)			− 0.11* (− 0.14, − 0.09)
Authoritarian Beliefs vs. Pandemic Threat			− 0.11* (− 0.15, − 0.07)
Authoritarian Beliefs vs. Threat (Health)			− 0.14* (− 0.17, − 0.11)
Authoritarian Beliefs vs. Threat (Economic)			− 0.02 (− 0.04, 0.003)
Authoritarian Beliefs vs. Threat (Self)			− 0.03* (− 0.05, − 0.005)
Authoritarian Beliefs vs. Threat (Society)			− 0.09* (− 0.12, − 0.07)
Health vs. Economic Threat			0.17* (0.14, 0.21)
Self vs. Society Threat			− 0.09* (− 0.12, − 0.06)

n = 1743. Shown are unstandardized direct effects (*p < 0.05, **p < 0.01, ***p < 0.001), simple indirect effects, pairwise contrasts of indirect effects, and 95% bias-corrected bootstrap confidence intervals in parentheses. All models control for age, sex, race/ethnicity, nativity status, southern residence, rural residence, college degree, employment, household income, financial strain, marital status, children, and religiosity

## Results

### Descriptive analyses

According to Table 1, the average respondent reported moderate levels of political conservatism and authoritarian beliefs, moderate to high levels of perceived pandemic threat (overall and with respect to health, economic status, self, and society), and high levels of empathic concern and healthy pandemic lifestyles. The average age of the sample was approximately 49 years, and more than half of the sample identified as female (52%). The race and ethnic composition of the sample included non-Hispanic Whites (67%), non-Hispanic Blacks (11%), Latinos (16%), and respondents of other races and ethnicities (6%). While few respondents reported being born in another country (8%) or living in a rural area (16%), it was more common for respondents to live in the South (33%). Over one-third of respondents reported having a four-year college degree or higher (36%), and over half of the sample reported being employed full- or part-time (62%). The average respondent reported an annual household income between $50,000 and $74,999 and low levels of financial strain. In terms of family characteristics, over half of the sample reported being married (54%), and few respondents reported the presence of a child under the age of 18 (17%). Finally, the average respondent exhibited low to moderate levels of religiosity.

### Regression analyses

In Table 2, political conservatism is inversely associated with empathic concern and perceived pandemic threat (overall and with respect to health, economic status, self, and society) and positively associated with authoritarian beliefs. In other words, respondents who score higher on political conservatism, averaged across indicators of Republican party identification, conservative political orientation, right-wing media consumption, and Trump support, also tend to report less concern for others, greater endorsement of authoritarian beliefs, and less concern about the pandemic. These associations persisted with adjustments for age, sex, race/ethnicity, nativity, southern residence, rural residence, education, employment status, household income, financial strain, marital status, the presence of children, and religiosity. According to the standardized regression coefficients, political conservatism is the strongest predictor of our proposed mediators. Perceived pandemic threat to economic status is the only exception. In this case, financial strain has the largest standardized coefficient, and political conservatism has the second largest. In terms of variance explained, R-squared values range from 0.08 (economic threat) and 0.12 (empathetic concern) to 0.25 (health threat) and 0.26 (authoritarianism).

In Table 3, political conservatism is inversely associated with healthy pandemic lifestyles across models. In other words, respondents who score higher on political conservatism also tend to report less engagement in pandemic lifestyles that comply with public health recommendations. Although 51% of the Model 1 (baseline model) association between political conservatism and healthy pandemic lifestyles is explained by the proposed mediators, the association persists in Model 9 (full model). We also see that empathetic concern and perceived pandemic threat (overall and with respect to health, economic status, self, and society) are positively associated with healthy pandemic lifestyles while authoritarian beliefs is inversely associated with healthy pandemic lifestyles. These patterns suggest that people who care less about the welfare of others, hold more authoritarian belief systems, and define the pandemic as less threatening to themselves and to the broader society also tend to report less engagement in healthy pandemic lifestyles. According to the standardized coefficients in Model 9, perceived pandemic threat is the strongest predictor of healthy pandemic lifestyles, and political conservatism is the second strongest predictor. We also note that political conservatism and age have the same standardized coefficient. In terms of variance explained, R-squared values are substantial, ranging from 0.27 to 0.43.

In Table 4, we regress our proposed mediators and healthy pandemic lifestyles on the individual items that make up the political conservatism index. We see that respondents who identify as Republican and score higher on conservative political orientation tend to report less empathetic concern, more authoritarian beliefs, less concern about the pandemic (overall and with respect to health, economic status, self, and society), and less healthy pandemic lifestyles. While voting for Trump in the 2020 Presidential election is associated with more authoritarian beliefs, less concern about the pandemic (overall and with respect to health, self, and society), and less healthy pandemic lifestyles, this particular political behavior is unrelated to empathetic concern and perceived economic threat. The patterns for Republican identification, conservative orientation, and Trump voter are remarkably consistent with the patterns observed for the full political conservatism index in Tables 2 and 3. Right-wing media consumption is an outlier. Although right-wing media consumption is associated with more authoritarian beliefs, this indicator of political conservatism is unrelated to empathic concern, pandemic threat, and pandemic lifestyle.

### Mediation analyses

The first column of Table 5 presents the unstandardized direct effects of political conservatism on healthy pandemic lifestyles. Each direct effect controls for background variables and the corresponding mediator. Consistent with the standardized direct effects presented in Table 3, political conservatism is inversely associated with healthy pandemic lifestyles while controlling for each of the proposed mediators. For example, the first direct effect of political conservatism on healthy pandemic lifestyles (b = − 0.32, p < 0.001) is net of background variables and empathy. The second direct effect is net of background variables and authoritarian beliefs.

The second column provides unstandardized indirect effects and 95% bootstrap confidence intervals (C.I.). Because none of the confidence internals contain zero, we observe statistically significant indirect effects of political conservatism on healthy pandemic lifestyles through empathic concern (I.E. = − 0.03, 95% C.I. − 0.04, − 0.02), authoritarian beliefs (I.E. = − 0.04, 95% C.I. − 0.06, − 0.02), overall perceived pandemic threat (I.E. = − 0.12, 95% C.I. − 0.14, − 0.10), threats to health (I.E. = − 0.18, 95% C.I. − 0.21, − 0.15), threats to economic status (I.E. − 0.02, 95% C.I. − 0.03, − 0.01), threats to self (I.E. = − 0.08, 95% C.I. − 0.09, − 0.06), and threats to society (I.E. = − 0.13, 95% C.I. − 0.15, − 0.11).

The last column includes pairwise contrasts of the indirect effects to formally assess the relative strength of our mediators. The C.I. for each point estimate indicates whether there is a difference between the two indirect effects. For example, the first point estimate (0.003, C.I. − 0.02, 0.03) compares the indirect effects for empathy and authoritarian beliefs. Because the C.I. contains zero, these indirect effects are likely comparable in magnitude. The sign of statistically significant point estimates indicates which indirect effect is larger. For example, because the sign for the contrast of the indirect effects for empathy and perceived pandemic threat is negative (− 0.10, C.I. − 0.13, − 0.08), the first indirect effect listed (empathy) is likely smaller. The indirect effect for empathy is also smaller than the indirect effect for threats to health (− 0.16, C.I. − 0.19, − 0.19), self (− 0.05, C.I. = − 0.07, − 0.03), and society (− 0.10, C.I. = − 0.13, − 0.08). The contrast of empathy and economic threat suggests that the indirect effects are likely comparable (− 0.001, C.I. = − 0.02, 0.01). The indirect effect for authoritarian beliefs is smaller than the indirect effects observed for pandemic threat (− 0.11, C.I. − 0.15, − 0.07), threats to health (− 0.14, C.I. − 0.17, − 0.11), threats to self (− 0.03, C.I. − 0.05, − 0.005), and threats to society (− 0.09, C.I. − 0.12, − 0.07). The contrast of authoritarian beliefs and economic threat suggests comparable indirect effects (− 0.02, C.I. − 0.04, 0.003). When we compared the indirect effects of different dimensions of pandemic threat, we observed that the indirect effect for threats to health was larger than the indirect effect for economic threats (0.17, C.I. 0.14, 0.21). We also found that the indirect effect for threats to self was smaller than the indirect effect for threats to society (− 0.09, C.I. − 0.12, − 0.06).

### Supplemental analyses

In supplemental analyses (not shown), we replicated our focal regressions with sample weights. Our results were also robust to the exclusion of individual index items (e.g., removing right-wing media consumption from the political conservatism index, the democracy and elections item from the authoritarian belief index, and hand sanitizing from the lifestyle index). The potential for multicollinearity was formally diagnosed by examining variance inflation factors (VIF) for each regression coefficient. VIFs above 10.00 are generally considered to indicate problematic multicollinearity [86]. Throughout our analyses, all VIFs were below 2.05. Although we are primarily interested in documenting the direct and indirect effects of political conservatism, we also tested whether the effects of political conservatism on the mediators and healthy pandemic lifestyles varied by region of residence and rurality. We tested 16 total interactions (conservatism*south and conservatism*rural when predicting the mediators and pandemic lifestyles). Only 3 of the 8 (37%) interactions between political conservatism and southern residence were different from zero. The inverse effects of political conservatism on empathy (b = 0.08, p < 0.05) and threats to health (b = 0.15, p < 0.05) and self (b = 0.11, p < 0.05) were weaker for respondents living in southern states. The effects of political conservatism on authoritarian beliefs, overall perceived threat, economic threats, and threats to society did not vary by region. All 8 interactions between political conservatism and rural residence were null. This suggests that the effects of political conservatism on the mediators and health lifestyles were comparable for respondents living in rural and non-rural areas.

## Discussion

In this paper, we developed and tested a mediation model to help explain *why* political conservatism is often associated with riskier pandemic lifestyles. We derived three hypotheses from our theoretical model. Our first hypothesis stated that the association between political conservatism and risky pandemic lifestyles would be mediated by lower levels of empathetic concern. Our second hypothesis predicted that the association between political conservatism and risky pandemic lifestyles would be mediated by higher levels of authoritarian beliefs. Our final hypothesis suggested that the association between political conservatism and risky pandemic lifestyles would be mediated by lower levels of perceived pandemic threat. Our core findings supported each of the three hypotheses by demonstrating that political conservatism is associated with less healthy pandemic lifestyles, at least in part, because political conservatives tend to be less empathetic, hold more authoritarian beliefs, and feel less threatened by the pandemic. In other words, political conservatives tend to engage in riskier pandemic lifestyles because they are less likely to care about the welfare of others, more likely to hold authoritarian beliefs, and less likely to perceive the pandemic as threatening to themselves (personal health and finances) and to the broader society (public health and the broader economy).

In our review of the pandemic literature, we could find only one mediation study that was relevant to our work. Using data collected from 8,800 registered voters in California, Shepherd et al. [17] reveal that trust in public health institutions mediates the effects of political worldview (party affiliation, political ideology, and Trump approval) on the perceived efficacy of public health prevention practices, but not the perceived threat of COVID-19. They also report that perceptions of the efficacy of staying home mediate the effect of political worldview on the frequency of leaving the house for work. Our study contributes to our empirical understanding of the processes underlying political variations in pandemic behavior by establishing the mechanisms of empathy, authoritarian beliefs, and pandemic threat (overall and with respect to health, self, and society) in a large national study of adults living in the United States. We advance health lifestyle theory by exploring pandemic lifestyles and addressing understudied political and ideological forces [24–27]. We verified that pandemic behaviors related to social distancing, hand sanitizing, mask wearing, and vaccination could load together on a single construct. We also found that pandemic lifestyles are socially patterned by a range of factors, including political conservatism, empathy, authoritarian beliefs, pandemic threat (overall and with respect to health, self, and society), age, sex, nativity, rurality, socioeconomic status, and family characteristics. Moving forward, it will be interesting to document the extent to which new pandemic health behaviors and lifestyles are retained as habitus.

In addition to these primary considerations, we would like to briefly discuss some of our secondary contributions. In support of previous research, we confirmed that political conservatism is associated with lower levels of empathy [28–35], higher levels of authoritarian beliefs [48–54], and lower levels of several dimensions of perceived pandemic threat [1, 13–15, 17, 59–63]. Our analyses of our disaggregated conservatism index are also informative. Despite all of the attention given to Trump supporters and right-wing media, we find that Republican party identification and conservative political orientation are the most consistent predictors of our focal mediators and pandemic lifestyles. Although voting for Trump is predictably associated with authoritarian beliefs, pandemic threat, and pandemic lifestyles, this political behavior is unrelated to empathy. While right-wing media consumption is associated with more authoritarian beliefs, it is unrelated to empathy, pandemic threat, and pandemic lifestyles. Even with all of the pandemic-related misinformation, disinformation, and conspiracy theories [4, 60, 87], right-wing media consumption is only indirectly associated with riskier pandemic lifestyles through the mechanism of authoritarian beliefs, not empathy or, perhaps most surprisingly, pandemic threat.

Our analyses indicate that perceived pandemic threat is the strongest mediator of political conservatism. With respect to pandemic threats, threats to health (personal and public) exhibited the strongest indirect effects while threats to economic status (personal finances and the broader economy) produced the weakest indirect effects. In fact, the indirect effects for threats to economic status were statistically indistinguishable from the indirect effects for empathy and authoritarian beliefs. The indirect effects for threats to health were stronger than the indirect effects for threats to economic status. The indirect effects for sociotropic threats to society (public health and the broader economy) were larger than the indirect effects for egocentric threats to self (personal health and finances). Our results for perceived pandemic threat are important because they highlight the unique impact of a top-down “discursive superstructure” [88, 89]. In the unique case of the pandemic, the messaging from political elites was sufficient to reverse the typical threat sensitivity of political conservatives. The more modest indirect effects associated with perceived economic threat are likely explained by Trump’s consistent messaging about the strength of the economy and conservative economic arguments against lockdown policies. Our results also tend to support the salience of sociotropic values over egocentric values for diverse political processes [90, 91].

Our analyses should be considered in the context of two central limitations. First and foremost, no causal inferences can be made because our analyses are based on a cross-sectional design. Although our proposed theoretical model suggests certain causal relationships, we fully acknowledge alternative theoretical models. We recognize that political ideology and “pro-social health behaviors” can be outcomes of biology (e.g., genetics), personality (e.g., perceived threat), moral foundations (e.g., harm and authority), and basic human values (e.g., universalism and conservation) [19, 46, 88–94]. These perspectives suggest that our proposed theoretical model could be reorganized to frame political conservatism as an outcome or mechanism of empathy (moral concern), authoritarian beliefs (value), and perceived pandemic threat (personality). For example, empathy and authoritarian beliefs could reasonably precede political conservatism in our data. All we know for sure is that our associations are consistent with similar associations published before the pandemic. However, pandemic threat is less likely to precede political conservatism because political conservatives tend to be less threatened by the pandemic. Before the pandemic, researchers argued that political conservatives were often motivated by threat and uncertainty to embrace authoritarianism and other conservative identities and ideologies. The logic of such “epistemic” and “existential” processes seems less applicable when political conservatives exhibit less threat sensitivity vis-à-vis the pandemic. As social scientists, we tend to favor social models (e.g., elite cues and political socialization) over personality models (e.g., pre-political psychological predispositions). This tendency is due to our training and expertise, not to any intentional exclusion of alternative models. Given that the associations in our proposed model are likely to be bidirectional or mutually reinforcing, we cannot exclude, theoretically or empirically, viable alternatives to our proposed model. In other words, our model is only one of many potential models. Second, although CHAPS is unique in its assessment of empathy and authoritarian beliefs, our measurements include only three-items. These indices are valid and reliable, but we acknowledge that more detailed assessments are available [45–47].

## Conclusions

Although our cross-sectional data cannot lead to any causal claims, we believe they offer new insights into the social epidemiology of infectious disease behavior. We are confident that the association between political conservatism and riskier pandemic lifestyles is at least partially mediated by the mechanisms of empathy, authoritarian beliefs, and pandemic threat. Of course, our analyses are contingent upon replication with longitudinal data and more comprehensive assessments of empathy and authoritarianism. Our work may also be extended through the exploration of additional mechanisms (e.g., trust in the institutions of government, science, and public health) and methodologies for assessing lifestyles (e.g., latent class analysis). The association between political conservatism and pandemic lifestyles may also vary according to theoretically relevant subgroups (e.g., political sophistication and level of perceived pandemic threat) and contexts (e.g., the unique political environments of states and countries and specific ideological conflicts) [95–97]. There is also the interesting question of whether the association between political conservatism and pandemic lifestyles could be suppressed by disgust sensitivity. If political conservatives tend to exhibit higher levels of disgust sensitivity (e.g., concern with diseases) [98, 99], they would likely engage in even riskier pandemic lifestyles in the absence of this particular motivation to avoid contagion. However, if we assume that disgust sensitivity presupposes threat perceptions, the fact that conservatives tend to be less concerned with the pandemic may limit the role of disgust sensitivity as a suppressor. A recent study by Gonzalez et al. [4, p. 2378] concluded that “because the coronavirus is a contagious infectious disease rather than a chronic disease that develops over the life course, systematic ideological resistance to public health recommendations is an immediate existential threat to society.” Understanding why and under what conditions political conservatives tend to engage in riskier pandemic lifestyles may eventually lead us to ways of identifying and overcoming widespread cultural barriers to critical pandemic responses.

## Data Availability

Raw data are not publicly available, but raw output and supplemental analyses are available upon request from the lead author.

## Code availability

Code is available upon request from the lead author.
